# Selective serotonin reuptake inhibitor use during pregnancy and maternal depression—a nationwide birth cohort study on risks to the mother and the newborn

**DOI:** 10.1016/j.ajogmf.2026.101910

**Published:** 2026-02-05

**Authors:** Heli Malm, Alan S. Brown, Keely Cheslack-Postava, Mika Gissler, David Gyllenberg, Emmi Heinonen, Susanna Hinkka-Yli-Salomaäki, Ian W. McKeague, Aleksi Tornio, Subina Upadhyaya, Andre Sourander

**Affiliations:** Research Centre for Child Psychiatry, Department of Child Psychiatry, University of Turku, INVEST flagship Centre, Turku, Finland (Malm, Gissler, Gyllenberg, Heinonen, Hinkka-Yli-Salomäaki, Upadhyaya, and Sourander); Teratology Information, Department of Emergency Medicine Services, Helsinki University and Helsinki University Hospital, Helsinki, Finland (Malm); Individualized Drug Therapy Research Program, Faculty of Medicine, University of Helsinki, Helsinki, Finland (Malm); Department of Clinical Pharmacology, Helsinki University and Helsinki University Hospital, University of Helsinki, Helsinki, Finland (Malm); Department of Psychiatry, New York State Psychiatric Institute, Columbia University Irving Medical Center, New York, NY (Brown and Cheslack-Postava); Department of Epidemiology, Columbia University, Mailman School of Public Health, New York, NY (Brown); Department of Data and Analytics, THL Finnish Institute for Health and Welfare, Helsinki, Finland (Gissler); Region Stockholm, Academic Primary Health Care Centre, Stockholm, Sweden (Gissler); Karolinska Institutet, Department of Molecular Medicine and Surgery, Stockholm, Sweden (Gissler); Department of Biostatistics, Columbia University, Mailman School of Public Health, New York, NY (McKeague); Institute of Biomedicine, University of Turku and Unit of Clinical Pharmacology, Turku University Hospital, Turku, Finland (Tornio).

**Keywords:** cohort study, gestational diabetes, maternal depression, pregnancy and neonatal outcomes, preterm birth, sibling study, SSRIs

## Abstract

**BACKGROUND:**

Maternal underlying depression may confound previously reported associations between selective serotonin reuptake inhibitor (SSRI) use and adverse pregnancy and neonatal outcomes.

**OBJECTIVE:**

To determine whether SSRI use during pregnancy is associated with an increased risk of pregnancy and neonatal complications after adjusting for indicators of maternal depression severity.

**STUDY DESIGN:**

This population-based birth cohort study used data from national registers in Finland and included 1272,587 singleton live births from 1996 to 2018. Pregnancy outcome of women with two or more SSRI purchases during pregnancy (*N*=19,020) were compared to women with a diagnosis of depression but no antidepressant use (*N*=19,625), and women who discontinued SSRIs before pregnancy (*n*=3145). Analyses included adjustment for several indicators of depression severity and within-family sibling comparisons.

**RESULTS:**

After adjusting for confounders and comparing to women with depression who did not use antidepressants, maternal SSRI use was associated with an increased risk of gestational diabetes (OR 1.14; 95% CI 1.07–1.22), while the risk of cesarean section (CS), late (32–36+6 weeks’ gestation) and very preterm birth (<32 weeks’ gestation), small for gestational age, and low and very low birth weight was lower. Among SSRI-exposed infants, risk of a low (<7) 5-minute Apgar score (OR 2.02; 95% CI 1.78–2.30), breathing problems (OR 1.61; 95% CI 1.48–1.75), and neonatal care unit (NCU) treatment (OR 1.23; 95% CI 1.16–1.31) was higher, whereas the risk of hospital stay at 7 days and major congenital anomalies was lower. Third-trimester exposure further increased the risk of a low 5-minute Apgar score (OR 3.44; 95% CI 2.93–4.04). After adjustment for indicators of depression severity, the increased risk of gestational diabetes persisted (OR 1.20; 95% CI 1.09–1.32), as did the lower risk of CS, very preterm birth, and low and very low birth weight, and the risks of a low 5-minute Apgar score, breathing problems, and NCU treatment remained higher. Compared to women who discontinued SSRI use before pregnancy, SSRI use was associated with lower risks of late preterm birth and low birth weight (OR 0.83; 95% CI 0.70–0.999 and OR 0.78; 95% CI 0.64–0.96, respectively), while the neonatal risks described above remained elevated. In the sibling-pair analysis, SSRI use was associated with an increased risk of gestational diabetes and neonatal complications other than malformations, including an increased risk of needing hospital stay at 7 days of age.

**CONCLUSION:**

SSRI use during pregnancy affects neonatal health beyond maternal depression by increasing symptoms related to delayed neonatal adaptation, although it may reduce the risk of preterm birth. The observed increase in the risk of gestational diabetes warrants further study.

## Tweetable statement

SSRI use during pregnancy affects neonatal health beyond maternal depression, increasing neonatal adaptation problems while potentially reducing preterm birth risk; increased gestational diabetes risk warrants further study.

## Introduction

Perinatal depression affects an estimated 10% to 15% of women, and moderate or severe symptoms often require antidepressant medication. Selective serotonin reuptake inhibitors (SSRIs) are the most used antidepressants during pregnancy, with 5% to 10% of pregnant women using them. Several studies on the safety of SSRIs during pregnancy have been published with conflicting results.^1–6^ Observational studies often have limited information on important covariates and confounders, including maternal depression and depression severity, potentially biasing the findings.^2^ While the risk of neonatal adaptation problems, including low Apgar scores and breathing difficulties, has often been reported to be increased in neonates prenatally exposed to SSRIs,^7,8^ recent research suggests that these issues are largely attributable to maternal depression itself.^2^ Research results regarding the risk of preterm birth have also been conflicting.^2,3,6^ While psychotherapy plays an important role in the treatment of depression, access to these therapies is often limited, creating situations where antidepressant treatment cannot be avoided. Safe treatment of maternal depression is important from a public health perspective. The aim of this study was to distinguish between the risks of SSRIs used during pregnancy and the risks associated with maternal depression regarding pregnancy and neonatal complications.

## Material and methods

This is a population-based birth cohort study, based on national registers in Finland. The study design has been described previously.^9^ Register data were linked using the unique personal identification numbers. The study was approved by the Ethics Committee of the Turku University Hospital and the Institutional Review Board of the New York State Psychiatric Institute. The Finnish Social and Health Data Permit Authority (FINDATA) gave their permission to use the register data in this study. The total sampling frame includes 1272,587 singleton live births between 1996 and 2018.

*The Medical Birth Register (MBR)* includes comprehensive information on maternal demographic characteristics, medical and reproductive history, health-related behaviors, diagnoses during pregnancy and delivery, and neonatal outcomes.

*The Hospital Discharge Register (HDR)* covers all hospital inpatient episodes in public and private institutions and outpatient public hospital visits since 1967, and all contacts in outpatient clinics since 1998. Psychiatric and somatic diagnoses made in primary care from 2011 onwards were collected from the *Register on Primary Health Care Visits*.

*The Drug Reimbursement Register (DRR)* contains data on reimbursed drug purchases, including the International Anatomic-Therapeutic-Chemical (ATC) classification code.^10^ Prescription-only medicines necessary for the treatment of an illness are reimbursed for all permanent residents in Finland. Information on major congenital anomalies was collected from the *Register for Congenital Malformations*. Sociodemographic background information and data from the father were obtained from *Statistics Finland* and the *Population Information System*.

### Exposure and comparison groups

*SSRI users* included women with two or more SSRI purchases (ATC code N06AB, including fluoxetine, citalopram, paroxetine, sertraline, fluvoxamine, escitalopram) from 30 days before pregnancy until the end of pregnancy.

*Women with depression but no antidepressants* included women diagnosed with depression or another psychiatric disorder related to depression or SSRI use in the HDR (footnote b, Table 1) from 1 year before pregnancy until discharge (≤3 weeks) from hospital after delivery, but no purchase of antidepressants or antipsychotics from 3 months before until the end of pregnancy.

The *Unexposed* group included women with neither purchase of antidepressants or antipsychotics at any time prior to or during pregnancy and no diagnoses of depression or psychiatric disorders related to depression or antidepressant use (footnote *c*, Table 1) at any time prior to or during pregnancy until discharge (≤3 weeks) from hospital after delivery.

A sensitivity analysis included women who discontinued SSRIs prior to pregnancy. This *SSRI discontinued group* included women who had purchase(s) of SSRIs 1 year before pregnancy until 3 months before pregnancy, but not during pregnancy. Within family comparisons included pregnancies of the same woman, either using or not using SSRIs during pregnancy.

### Covariates

Covariates were obtained from the registers and included maternal, neonatal, and family characteristics (Table 1 and Supplemental Table 1).

### Outcome variables

**FIGURE 1:**
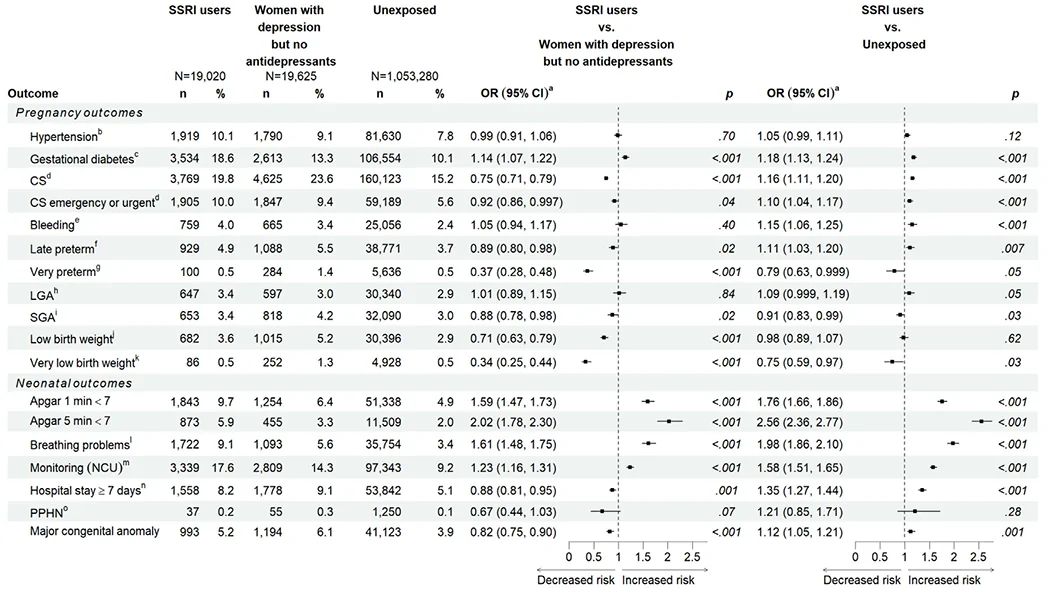
Pregnancy and neonatal complications, comparisons between SSRI users, women with depression but no antidepressant use, and unexposed. *SSRI users:* women with two or more SSRI purchases (ATC code N06AB; including fluoxetine, citalopram, paroxetine, sertraline, fluvoxamine, escitalopram) from 30 days before pregnancy until the end of pregnancy. *Women with depression but no antidepressants:* pregnancies of women diagnosed with depression or another psychiatric disorder related to depression or SSRI use from 1 year before pregnancy until discharge (≤3 weeks) from hospital after delivery, and with no maternal purchases of antidepressants or antipsychotics from 3 months before until the end of pregnancy. Unexposed: no maternal prescriptions purchased for antidepressants or antipsychotics at any time prior to or during pregnancy, nor any diagnoses of depression or psychiatric disorders related to depression or antidepressant use at any time prior to or during pregnancy until discharge (≤3 weeks) from hospital after delivery. ^a^Adjusted for variables associated with exposure and outcome at *P*<.1 (Supplemental Table 1). ^b^Hypertension of pregnancy/preeclampsia: ICD-10 010-016. ^c^Gestational diabetes: ICD-10 024.4; ICD-10 024.9. ^d^CS, cesarean section. ^e^Bleeding during or after delivery: during delivery ICD-10 067 (ICD-10 067.0; 67.8; 67.9); after delivery ICD-10 072 (ICD-10 072.0–72.3). ^f^Late preterm 32 to 36+6 weeks. ^g^Very preterm <32 weeks. ^h^Large for gestational age (birth weight more than two standard deviations above national standards for sex and length of gestation). ^i^Small for gestational age (birth weight less than two standard deviations below national standards for sex and length of gestation). ^j^Low birth weight, <2500 g. ^k^Very low birth weight (<1500 g). ^l^Neonatal breathing problems; ICD-10 P22. ^m^NCU, neonatal care or intensive care unit. ^n^Infant at hospital at 7 days of age. ^o^Persistent pulmonary hypertension of the newborn; ICD-10 P29.31. Source: Malm. Selective serotonin reuptake inhibitor use during pregnancy and maternal depression. Am J Obstet Gynecol MFM 2026.

The outcome variables were derived from existing literature.^1–8^ The outcome variables for pregnancy included hypertension of pregnancy/preeclampsia; gestational diabetes; mode of delivery (vaginal/cesarean section, CS); emergency or urgent CS; bleeding during or after delivery; late preterm birth 32 to 36+6 weeks; very preterm birth <32 weeks; large for gestational age (LGA, birth weight more than two standard deviations above national standards for sex and length of gestation); small for gestational age (SGA, birth weight less than two standard deviations below national standards for sex and length of gestation); low birth weight (<2500 g); and very low birth weight (<1500 g); outcome definitions in footnotes *b* to *k*, Figure 1.

The neonatal outcome variables included 1 and 5-minute Apgar score <7; neonatal breathing problems; need for monitoring in neonatal care or intensive care unit (NCU); hospital stay of infants at 7 days of age; pulmonary hypertension; and major congenital anomaly (footnotes *l*–*o* Figure 1).

### Maternal depression severity

**FIGURE 2:**
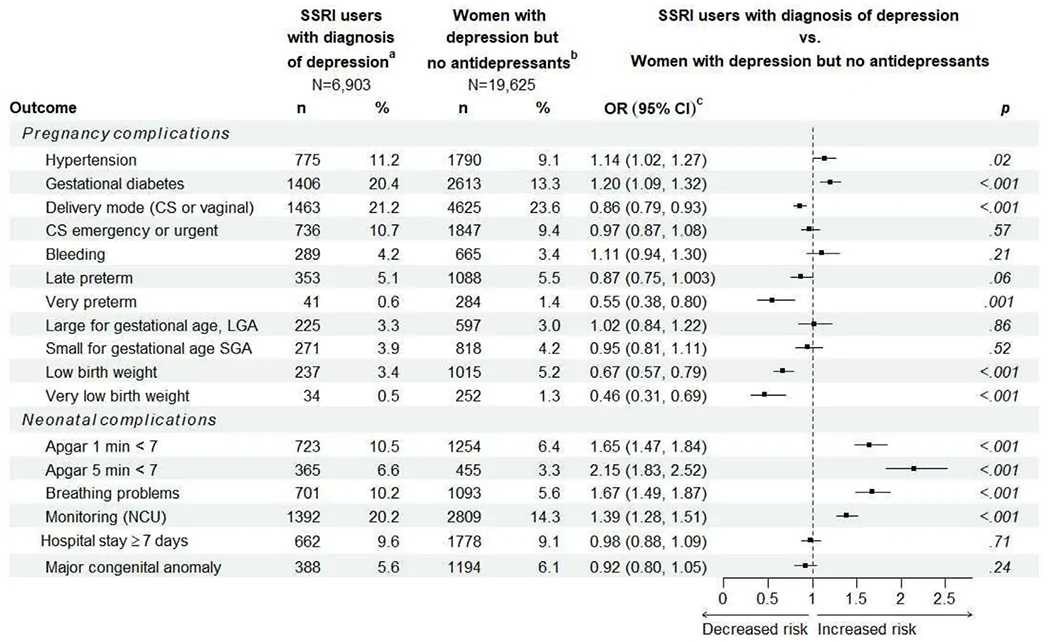
Comparisons between SSRI users with a diagnosis of depression and women with a diagnosis of depression but no antidepressant use, adjusted for indicators of maternal depression severity. ^a^Women with two or more SSRI purchases from 30 days before pregnancy until the end of pregnancy *and* a diagnosis of depression or another psychiatric disorder related to depression or SSRI use from 1 year before pregnancy until discharge (≤3 weeks) from hospital after delivery. ^b^Women diagnosed with depression or another psychiatric disorder related to depression or SSRI use from 1 year before pregnancy until discharge (≤3 weeks) from hospital after delivery, and with no maternal purchases of antidepressants or antipsychotics from 3 months before until the end of pregnancy. ^c^Adjusted for variables associated with exposure and outcome at *P*<.1 (Supplemental Table 1). Also adjusted for indicators of maternal depression severity: maternal age of depression onset (≤19, 20–29, 30–39, ≥40); category of diagnosis occurring 1 year before pregnancy until discharge (3-group hierarchical mutually exclusive variable: Nonaffective psychoses and transient psychoses: ICD-9 295, 297, 2988A, 2989X, 3013C, ICD-10: F20–F29; mood disorders: ICD-9 296, 3004A, ICD-10: F30–F39; and neurotic, stress-related and somatoform disorders: ICD-9 3000–3003, 3006–3009, 3078A, 3090A, 3092C-E, 3098A, 3098X, 3099X, ICD-10 F40–F48); hospitalization for depression severe (>2 inpatient hospitalizations over the illness course), moderate (at least 1 inpatient hospitalization over the illness course), and mild (no hospitalizations); and intentional self-harm requiring hospital treatment 1 year before pregnancy until discharge (≤3 weeks) from hospital after delivery: ICD-10 codes X60-X84, Z72.8, Z91.5 or ICD-9 codes E950-E959, V156, V658. Source: Malm. Selective serotonin reuptake inhibitor use during pregnancy and maternal depression. Am J Obstet Gynecol MFM 2026.

Indicators of maternal depression severity were obtained from the HDR, including information on the age of depression onset, specific psychiatric diagnoses, number of hospitalizations for depression, and diagnoses of intentional self-harm (footnote c,Figure 2).

### Statistical analysis

Covariates considered as potential confounders were tested for associations with exposure groups in the total population using the chi-square test or ANOVA. Those variables associated with both exposure and outcomes at *P* <.1 were included in the adjusted models. Fewer than 5% of observations were missing information on covariates, and thus, complete case analysis was performed (models included observations with complete data on all covariates).

The associations between exposure group and outcomes were examined in covariate-adjusted logistic regression models using generalized estimating equations (GEE). To account for correlation between pregnancies in the same family, data from siblings were treated as clusters, and robust standard errors were estimated. The impact of the gestational timing of SSRI exposure on pregnancy and neonatal outcomes was analyzed by restricting the exposed group to individuals with at least one SSRI purchase during the first and second trimesters or the third-trimester.

Sensitivity analyses included adjustment for indicators of maternal severity of depression, and comparison of pregnancies with maternal use of SSRIs to pregnancies where the woman was prescribed, but discontinued use of an SSRI prior to pregnancy.

To address potential confounding by family-level factors, outcomes of discordantly exposed siblings were compared using GEE models.

To further assess the impact of family characteristics on the observed relationships, the association of paternal SSRI exposure (defined as two or more SSRI purchases during the woman’s pregnancy) vs no paternal SSRI exposure—and pregnancy and neonatal outcomes was analyzed by comparison to pregnancies among those unexposed to maternal antidepressant use or depression. Analyses were performed using SAS version 9.4 (SAS Institute Inc). Statistical significance was set at *P*<.05.

## Results

SSRIs were purchased in a total of 30,815 (2.4%) pregnancies, and in 19,020 pregnancies, the woman made two or more SSRI purchases. The comparison group of women with depression but no antidepressants included 19,625 pregnancies, and the group of women unexposed to depression or antidepressants included 1053,280 pregnancies. Maternal and family characteristics of the exposure groups are shown in Table 1, and association of these covariates to the outcome variables are presented in Supplemental Table 1.

### Comparisons between SSRI users, women with depression but no antidepressants, and unexposed.

After adjusting for confounders, maternal SSRI use was associated with an increased risk of gestational diabetes (OR 1.14; 95% CI 1.07–1.22), whereas the risks of several other pregnancy complications—including CS, late and very preterm birth, SGA, and low and very low birth weight—were lower (Figure 1). Neonatal risks, including low (<7) 1- and 5-minute Apgar scores, breathing problems, and NCU treatment, were higher among SSRI-exposed infants, whereas the risks of hospital stay at 7 days of age and major congenital anomalies were lower. Compared to unexposed pregnancies, SSRI use was associated with a modestly increased risk of several pregnancy complications, whereas the risk of SGA and very low birth weight was lower. The risk of neonatal complications, other than persistent pulmonary hypertension of the newborn (PPHN), was increased (Figure 1). In analyses by individual SSRI, use of fluoxetine, citalopram, and escitalopram was associated with an increased risk of gestational diabetes, while escitalopram was also associated with bleeding during or after delivery. No other major differences between individual SSRIs were observed. Each SSRI was associated with an approximately twofold increase in the risk of a low 5-minute Apgar score (Supplemental Table 2).

*In trimester-specific analyses* that included women using SSRIs during the first and second trimesters, and comparing to women with depression but no antidepressants, the risk of very preterm birth was nearly 60% lower; however, the previously observed lower risks of late preterm birth, SGA, and hospital stay at 7 days were no longer evident among SSRI users (Supplemental Table 3). In analyses of maternal SSRI use during the third-trimester, restricted to full-term deliveries and neonatal outcomes other than congenital anomalies, maternal SSRI use was associated with an increased risk of neonatal complications other than PPHN, including a more than threefold risk of a low 5-minute Apgar score (Table 2).

*After adjustment for indicators of depression severity* and comparing SSRI users with a diagnosis of depression (*n*=6903) to women with depression who did not use antidepressants, the risk of hypertensive disorders of pregnancy/preeclampsia was elevated, and the increased risk of gestational diabetes persisted (OR 1.20; 95% CI 1.09–1.32). The lower risks of CS, very preterm birth, and low and very low birth weight also remained. Neonatal risks—other than hospital stay at 7 days of age and major congenital anomalies—remained elevated (Figure 2).

### Comparisons between SSRI users and women who discontinued SSRI use before pregnancy.

**Figure 3:**
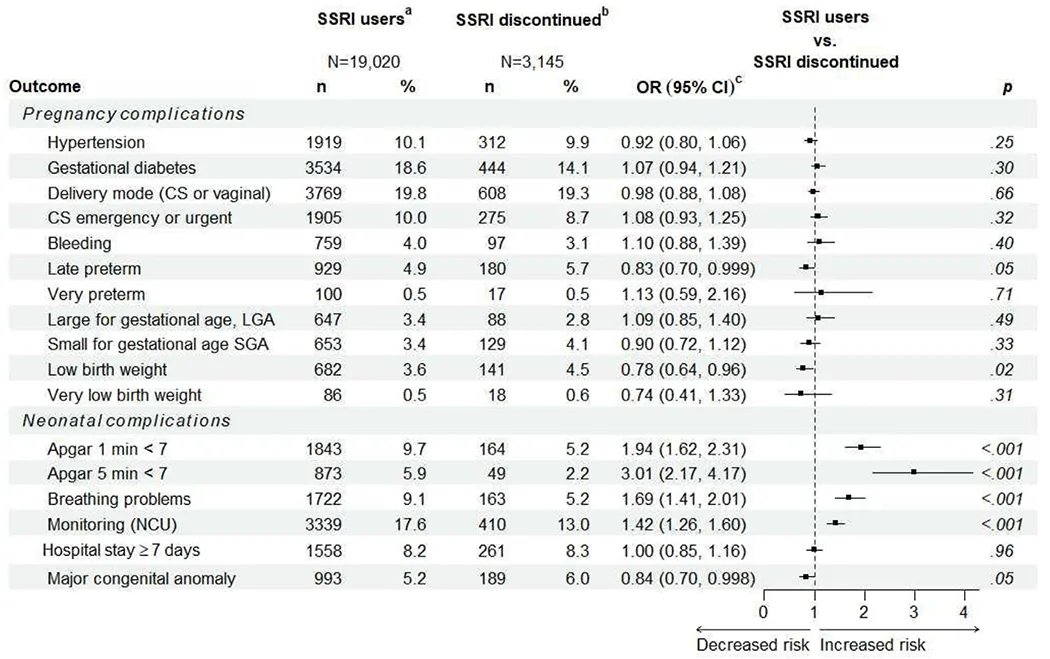
Comparisons between SSRI users and women who discontinued SSRI use before pregnancy. ^a^Based on at least two purchases of SSRIs (ATC code N06AB; including fluoxetine, N06AB03; citalopram, N06AB04; paroxetine, N06AB05; sertraline, N06AB06; fluvoxamine, N06AB08; escitalopram, N08AB10). ^b^Women who had purchase(s) of SSRIs 1 year before pregnancy until 3 months before pregnancy, but not during pregnancy. ^c^Adjusted for variables associated with exposure and outcome at *P*<.1 (Supplemental Table 1). Source: Malm. Selective serotonin reuptake inhibitor use during pregnancy and maternal depression. Am J Obstet Gynecol MFM 2026.

SSRI use during pregnancy was not associated with an increased risk of gestational diabetes but was associated with lower risks of late preterm birth and low birth weight (OR 0.83; 95% CI 0.70–0.999 and OR 0.78; 95% CI 0.64–0.96, respectively). The risks of low 1- and 5-minute Apgar scores, breathing problems, and NCU treatment remained higher (Figure 3).

### Sibling analyses.

**FIGURE 4:**
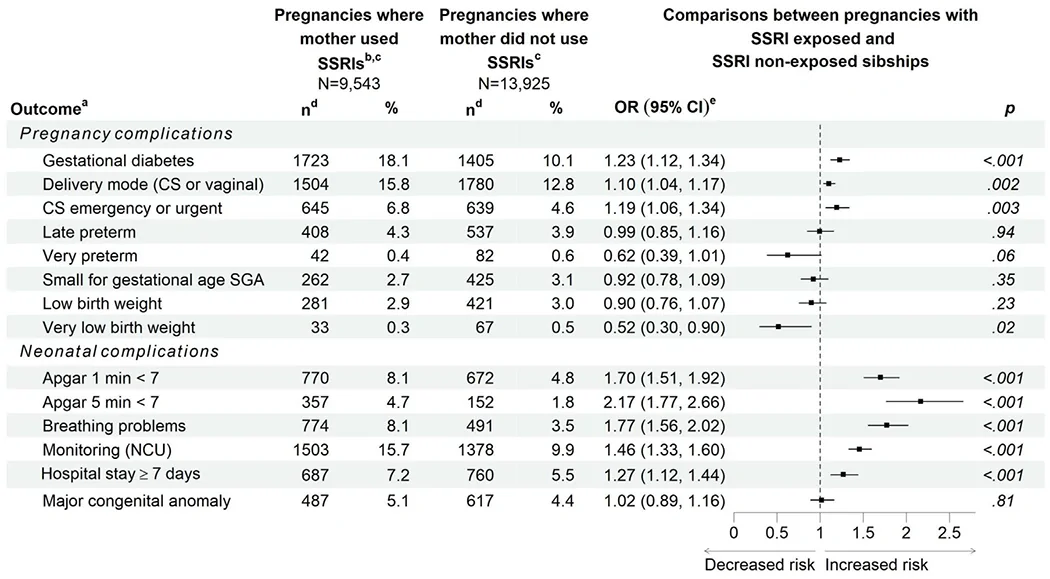
Pregnancy and neonatal complications within sibships, discordant to maternal SSRI use during pregnancy. ^a^Including outcomes with significant results in full sample analysis. ^b^Based on at least 2 purchases of SSRIs. ^c^From families with discordant outcomes. ^d^Case counts of siblings based on discordant families. ^e^Adjusted for variables associated with exposure and outcome at *P*<.1 (Supplemental Table 1). Source: Malm. Selective serotonin reuptake inhibitor use during pregnancy and maternal depression. Am J Obstet Gynecol MFM 2026.

The risk of gestational diabetes and CS was higher in SSRI-exposed pregnancies, whereas the risk of very preterm birth and very low birth weight were lower; however, the lower risk of very preterm birth did not reach statistical significance (Figure 4). Infants prenatally exposed to SSRIs had higher risks of low 1- and 5-minute Apgar scores, neonatal breathing problems, NCU treatment, and hospital stay at 7 days (Figure 4).

Paternal use of SSRIs was not associated with an increased risk of any of the outcomes (Supplemental Table 4).

### Comparisons between women with depression but no use of antidepressants and the unexposed

Comparing women with depression but no use of antidepressants to the unexposed, the risk of most pregnancy complications—excluding gestational diabetes, LGA, and SGA—was increased. The risk of very preterm birth was increased more than twofold (OR 2.15; 95% CI 1.86–2.48). The risk of all neonatal problems was higher, including a low 5-minute Apgar score (OR 1.26; 95% CI 1.13–1.41), and the risk for PPHN was nearly twofold (OR 1.80; 95% CI 1.34–2.41). The risk of major congenital anomalies was also elevated (OR 1.36; 95% CI 1.28–1.45) (Supplemental Table 5).

## Comment

### Principal findings

Using national register data, we found an increased risk of gestational diabetes among women using SSRIs during pregnancy; however, this association was not consistent across all comparisons. Maternal SSRI use was associated with a lower risk of very preterm birth and a higher risk of complications related to neonatal adaptation. Maternal depression itself was associated with several pregnancy and neonatal complications, including major congenital anomalies.

### Results in the context of what is known

The prevalence of gestational diabetes varies depending on the approach to screening and diagnosis, with a global estimate of around 14%.^11^ General screening for risk factors associated with gestational diabetes is recommended already when planning for pregnancy.^12^ The use of antidepressants, in general, has been associated with an increased risk of type 2 diabetes in nonpregnant individuals; however, this association is likely confounded by underlying depression.^13^ Studies involving pregnant women taking antidepressants have reported conflicting results suggesting that the risk may differ between antidepressants with different pharmacological properties.^14,15^ Previous studies that accounted for maternal underlying depression did not find an increased risk of gestational diabetes in SSRI users, but the available information remains limited.^14,16–18^

We observed an increased risk of gestational diabetes among SSRI users, and this association persisted after adjustment for several indicators of maternal depression severity and in sibling comparisons. However, the magnitude of risk was similar among SSRI users and women who discontinued SSRI use prior to pregnancy. Current and former SSRI users may share characteristics not captured in registry data, which could act as confounders and help explain the observed findings.^19^ It is also possible, however, that SSRIs induce metabolic changes that persist beyond treatment discontinuation. In Finland, national screening and diagnostic guidelines for gestational diabetes were updated in 2008 and currently provide free screening to all expectant mothers at risk.^20^ If a true finding, this may explain why we did not observe an increased risk of pregnancy complications typically associated with gestational diabetes.^19,21^ While this study focused on pregnancy and neonatal complications, prenatal exposure to maternal hyperglycemia also predisposes offspring to long-term health problems, including childhood obesity and neuropsychiatric disorders.^22^

Animal studies suggest that prenatal SSRI exposure may reduce birth weight and shorten gestation, potentially due to increased maternal serotonin leading to uterine and placental vasoconstriction and impaired perfusion.^23^ Similarly, a meta-analysis in humans reported an increased risk of low birth weight, although few included studies adjusted for depression severity.^24^ In contrast, we observed no increased risk of SGA or low birth weight among SSRI users; rather, the risks of low and very low birth weight were lower in most comparisons.

The risk of late preterm birth was 10% lower, and the risk of very preterm birth was over 60% lower in SSRI users compared to women with depression but no antidepressants. Adjusting further for indicators of maternal depression severity, the risk of very preterm birth in SSRI users remained 45% lower. These findings confirm the findings from our previous study including register data through years 1996 to 2010^6^ but challenge several recent studies reporting an increased risk of preterm birth associated with SSRI use.^2–4^ Observational studies often have limitations, including potential exposure mis-classification and residual confounding by maternal depression severity and family-related factors, which may be reflected in the results of meta-analyses.^3^ In the present study, maternal depression was associated with a 25% increased risk of late preterm birth, and with a more than twofold increased risk of very preterm birth when compared to the unexposed. In Finland, all pregnant women have access to scheduled follow-up at outpatient maternity clinics, free of charge, and it is unlikely that depression or use of antidepressants would have an impact on access to perinatal care to the extent that could be reflected in our findings.

Our results are consistent with a recent study from a large population-based cohort in Northern California that adjusted for maternal depression and anxiety and reported approximately a twofold increase in the odds of delayed neonatal adaptation when SSRIs were used during the latter half of pregnancy.^8^ Our findings suggest a direct impact of SSRIs on neonatal health beyond the effects of maternal depression. This association is biologically plausible, as SSRIs cross the placenta and may affect multiple organs by increasing serotonergic activity, including the lungs and the central nervous system, thereby predisposing neonates to toxicity.^25–27^

SSRI use has previously been associated with an increased risk of postpartum bleeding^28–30^; however, we did not observe an elevated risk. We observed a small increased risk of hypertension/ preeclampsia among SSRI users, consistent with previous studies^29,31^ but this association was evident only when comparing SSRI users with women with depression who did not use antidepressants and after adjustment for indicators of depression severity. The results highlight the complex interplay between SSRI use, maternal depression, and pregnancy outcomes, emphasizing the need for further investigation into the implications of serotonin modulation on conditions like preeclampsia.

### Clinical implications

These results provide reassurance that SSRI use during pregnancy does not increase the risk of preterm birth and may even be associated with a reduced risk of preterm birth when treating maternal depression. Given the increased risk of problems related to neonatal adaptation, neonatal monitoring remains important. The observed increased risk of gestational diabetes among SSRI users warrants further investigation.

### Research implications

We suggest that future research should aim to replicate the findings regarding the increased risk of gestational diabetes in SSRI users across different countries and populations, and to examine whether this risk extends to other classes of antidepressants.

### Strengths and limitations

The study’s primary strength lies in its large, population-based national cohort, which provides comprehensive data on pregnancies, mothers, fathers, and siblings. The MBR and DRR data are practically complete, and the HDR data have been validated for psychiatric diagnoses.

To minimize bias related to drug compliance, only mothers with more than one SSRI prescription during pregnancy were included. We included several comparison groups and a sibling control group to account for familial and genetic factors and included data on paternal antidepressant use during the mother’s pregnancy to further mitigate family-related confounding. While we adjusted for several indicators of maternal depression severity, depression severity cannot be fully evaluated by using only register data, as depression symptoms were not measured directly. As in all observational studies, our findings indicate associations rather than causation, and residual confounding remains a concern.

## Conclusions

SSRI use during pregnancy has a direct impact on neonatal health beyond the effects of maternal depression. However, SSRI use does not increase the risk of preterm birth and may even be beneficial in reducing that risk when treating maternal depression. The observed increased risk of gestational diabetes warrants further study.

## Supplementary Material

1

2

Supplementary material associated with this article can be found in the online version at doi:10.1016/j.ajogmf.2026.101910.

## Figures and Tables

**TABLE 1:** Maternal, neonatal, and family characteristics tested as covariates by the three-class exposure status

Characteristics	SSRI users^a^ *N*=19,020		Women with depression but no antidepressants^b^ *N*=19,625		Unexposed *N*=1053,280^c^		
	*N*	%	*N*	%	*N*	%	*P* value
*Offspring sex (male)*	9745	51.24	10,051	51.22	539,168	51.19	.990
*Maternal age, y*							<.001
≤19	480	2.52	1582	8.06	24,449	2.32	
20–29	8478	44.57	9396	47.88	502,589	47.72	
30–39	9169	48.21	7879	40.15	491,430	46.66	
≥40	893	4.70	768	3.91	34,812	3.31	
*Paternal age, y* ^d^							<.001
≤19	187	1.01	574	3.06	7801	0.75	
20–29	6262	33.91	7472	39.84	359,433	34.65	
30–39	9383	50.81	8449	45.05	553,475	53.35	
≥40	2635	14.27	2261	12.05	116,667	11.25	
*Urbanicity* ^d^							<.001
Urban	12,895	67.84	13,961	71.19	700,510	66.62	
Semi-urban	3186	16.76	2999	15.29	176,676	16.80	
Rural	2926	15.39	2652	13.52	174,280	16.57	
*Marital status*							<.001
Unmarried (vs married/in a relationship or cohabitation)	2646	13.91	3448	17.57	93,417	8.87	
*Maternal socioeconomic status*							<.001
Upper white-collar workers	2241	11.78	2022	10.30	138,880	13.19	
Lower white-collar workers	5210	27.39	4371	22.27	254,772	24.19	
Blue collar workers	3216	16.91	2929	14.92	130,727	12.41	
Others^e^	2360	12.41	2098	10.69	64,660	6.14	
Missing	5993	31.51	8205	41.81	464,241	44.08	
*Paternal socioeconomic status*							<.001
Upper white-collar workers	2663	14.00	2276	11.60	148,086	14.06	
Lower white-collar workers	2852	14.99	2510	12.79	141,028	13.39	
Blue collar workers	5996	31.52	5384	27.43	233,125	22.13	
Others	3437	18.07	3489	17.78	117,648	11.17	
Missing	4072	21.41	5966	30.40	413,393	39.25	
*Maternal education*							<.001
Basic education (≤9 y)	4050	21.29	5643	28.75	148,323	14.08	
Secondary education (10–12 y)	8842	46.49	8057	41.05	419,839	39.86	
Tertiary education (13 y or more)	6128	32.22	5925	30.19	485,118	46.06	
Paternal education^d^							<.001
Basic education (≤9 y)	4017	21.75	4851	25.86	186,514	17.98	
Secondary education (10–12 y)	9497	51.43	9200	49.05	495,548	47.77	
Tertiary education (13 y or more)	4953	26.82	4705	25.09	355,314	34.25	
*One or more prior deliveries (vs none)* ^d^	11,410	60.03	10,350	52.77	617,701	58.70	<.001
*Prenatal maternal smoking* ^d^	5232	28.40	5140	27.10	135,086	13.19	<.001
*ART use, current pregnancy*	559	2.94	596	3.04	28,213	2.68	<.001
*Maternal prepregnancy BMI* ^f^							<.001
<18.5	540	2.84	768	3.91	22,313	2.12	
18.5–24.9	8415	44.24	8709	44.38	389,796	37.01	
25–29.9	3939	20.71	3289	16.76	133,978	12.72	
≥30	3166	16.65	2069	10.54	72,380	6.87	
Missing	2960	15.56	4790	24.41	434,813	41.28	
*Prepregnancy diabetes* ^g^	451	2.37	423	2.16	12,113	1.15	<.001
*Mother’s chronic diseases* ^h^	10,031	52.74	10,500	53.50	402,004	38.17	<.001
*Number of hospitalizations* ^i^							<.001
0	9037	47.51	9597	48.90	713,648	67.75	
1	8478	44.57	8251	42.04	310,699	29.50	
2	1121	5.89	1287	6.56	24,516	2.33	
≥3	384	2.02	490	2.50	4417	0.42	
Teratogen exposure^j^	375	1.97	248	1.26	5154	0.49	<.001
Anxiolytic/sedative exposure^k^	3555	18.69	1045	5.32	4668	0.44	<.001
Antiepileptic exposure^l^	563	2.96	330	1.68	2308	0.22	<.001
*Preterm birth in previous pregnancies*							<.001
Preterm birth, <37 wk	670	3.52	597	3.04	24,135	2.29	
Very preterm birth, <32 wk	109	0.57	156	0.79	3467	0.33	
*Hypertension/preeclampsia in previous pregnancies*	1601	8.42	1155	5.89	61,084	5.80	<.001
*Gestational diabetes in previous pregnancies*	1478	7.77	966	4.92	42,941	4.08	<.001
	**Mean**	**SD**	**Mean**	**SD**	**Mean**	**SD**	
***Birth year***	2010.11	5.17	2009.20	5.96	2006.26	6.53	<.001
***Maternal age***	30.00	5.62	28.46	6.23	29.56	5.32	<.001
***Paternal age***	32.48	6.69	31.18	7.09	32.08	6.19	<.001

a Based on at least 2 purchases of SSRIs (ATC code N06AB; including fluoxetine, N06AB03; citalopram, N06AB04; paroxetine, N06AB05; sertraline, N06AB06; fluvoxamine, N06AB08; escitalopram, N08AB10).;

b Diagnosis with: ICD-10 F20 to F48; ICD-9 295, 297, 2988A, 2989X, 3013C, 296, 3004A, 3000 to 3003, 3006 to 3009, 3078A, 3090A, 3092C-E, 3098A, 3098X, or 3099X from 1 year before pregnancy until discharge (≤3 weeks) from hospital after delivery, and with no maternal purchases of antidepressants (ATC codes N06A, N06CA) or antipsychotics (N05A) from 3 months before until the end of pregnancy.;

c No maternal prescriptions purchased for antidepressants or antipsychotics (N06A, N06CA, N05A) at any time prior to or during pregnancy and no diagnoses of depression or psychiatric disorders related to depression or SSRI use (ICD-10 F20–F48; ICD-9 295, 297, 2988A, 2989X, 3013C, 296, 3004A, 3000–3003, 3006–3009, 3078A, 3090A, 3092C-E, 3098A, 3098X, 3099X; ICD-8 295–300) at any time prior to or during pregnancy until discharge (≤3 weeks) from hospital after delivery.;

d Information missing for: paternal age and education, *n*=17,326; urbanicity, *n*=1840; prior births, *n*=1058; prenatal maternal smoking, *n*=30,385.;

e Students, housewives, entrepreneurs, or unemployed persons.;

f Available from 2004 onwards.;

g Including: ICD-10 E10-E14 recorded before LMP; ICD-10 024.0, 024.1, 024.2, 024.3; ICD-9 250 or ICD-8 250 recorded before LMP; Special Reimbursement Register 103, 171, 177, 215, 285 recorded before LMP.;

h Other chronic diseases ever recorded in the HDR or Special Reimbursement Register (see Supplemental Appendix 1).;

i Number of hospitalizations (other than psychiatric) from the beginning of pregnancy until delivery.;

j Maternal purchase any time during pregnancy or 1 month prior to pregnancy of: misoprostol (Anatomic-Therapeutic-Chemical, ATC, code A02BB01), warfarin (B01AA03), agents acting on the renin-angiotensin system (C09), etretinate (D05BB01), acitretin (D05BB02), isotretinoin (D10BA01), alitretinoin (D11AH04), carbimazole (H03BB01), antineoplastic agents (L01), mycophenolic acid (L04AA06), leflunomide (L04AA13), teriflunomide (L04AA31), thalidomide (L04AX02), methotrexate (L04AX03), lenalidomide (L04AX04), pomalidomide (L04AX06), misoprostol + diclofenac (M01AB55), ergot alkaloids (N02CA), valproic acid (N03AG01), carbamazepine (N03AF01), phenytoin and derivatives (N03AB), topiramate (N03AX11), or lithium (N05AN01).;

k Maternal purchase of anxiolytics (ATC N05B) and/or sedatives (N05C), any time during pregnancy or 1 month before pregnancy.;

l Maternal purchase of antiepileptic drugs (ATC N03; excluding those listed in “teratogens”), any time during pregnancy or 1 month before pregnancy.

Source: Malm. Selective serotonin reuptake inhibitor use during pregnancy and maternal depression. Am J Obstet Gynecol MFM 2026.

**TABLE 2:** Neonatal complications and third-trimester SSRI use

Outcome	SSRI use in 3rd trimester^a^ *N*=12,186		Women with depression but no antidepressants *N*=18,253		SSRI use in 3rd trimester vs women with depression but no antidepressants		
	*n*	%	*n*	%	OR^b^	95% CI	*P*
Apgar 1 min <7	1251	10.3	932	5.1	2.14	1.95–2.35	<.001
Apgar 5 min <7	593	6.1	261	2.0	3.44	2.93–4.04	<.001
Breathing problems^c^	1089	8.9	635	3.5	2.54	2.28–2.83	<.001
Monitoring (NCU)	2014	16.5	1907	10.4	1.62	1.51–1.75	<.001
Hospital stay ≥7 d^d^	781	6.4	1008	5.6	1.15	1.04–1.28	.009
PPHN^e^	26	0.2	27	0.1	1.41	0.80–2.47	.23

Comparisons to women with depression but no antidepressants. Analyses restricted to full-term (≥37 gestational weeks) infants. Major congenital anomalies not analyzed because the exposure window does not cover first trimester.

*NCU*, neonatal care or intensive care unit.

a At least one SSRI purchase during the third-trimester.;

b Adjusted for variables associated with exposure and outcome at *P*<.1 (Supplemental Table 1).;

c Neonatal breathing problems; ICD-10 P22.;

d Infant at hospital at 7 days of age.;

e Persistent pulmonary hypertension of the newborn; ICD-10 P29.31.

Source: Malm. Selective serotonin reuptake inhibitor use during pregnancy and maternal depression. Am J Obstet Gynecol MFM 2026.

## AJOG MFM at a Glance

### Why was this study conducted?

- Maternal underlying depression may confound the previously reported associations between SSRI use and adverse pregnancy and neonatal outcomes.

### Key findings

- Compared to women with depression who did not use antidepressants, and after adjustment for several indicators of depression severity, maternal SSRI use was associated with a lower risk of very preterm birth but a higher risk of gestational diabetes and complications related to neonatal adaptation.

### What does this study add to what is already known?

- SSRI use during pregnancy affects neonatal health beyond maternal depression by increasing symptoms related to delayed neonatal adaptation.
- Importantly, treating maternal depression with SSRIs may be beneficial in reducing the risk of preterm birth.
- The finding of an increased risk of gestational diabetes warrants further study.
