# Supplementary material for: Selective serotonin reuptake inhibitor use during pregnancy and maternal depression—a nationwide birth cohort study on risks to the mother and the newborn
Source: Am J Obstet Gynecol MFM. Author manuscript; Available in PMC 2026 Aug 21. (PMC13494963; doi:10.1016/j.ajogmf.2026.101910)
Supplement: Supplementary file 1 [file NIHMS2191537-supplement-2.docx]

**Supplemental Table 1.**  Association of covariates to the outcome variables under study.

| Covariate associated with exposure at P<0.1 | Outcome | | | | | | | | | | | | | | | | | |
| --- | --- | --- | --- | --- | --- | --- | --- | --- | --- | --- | --- | --- | --- | --- | --- | --- | --- | --- |
|  | Hypertension | Gestational diabetes | Delivery mode (CS or vaginal) | CS emergency or urgent | Bleeding | Late preterm | Very preterm | LGA | SGA | Low birth weight | Very low birth weight | Apgar 1 min | Apgar 5 min | Breathing problems | Monitoring (NCU) | Hospital stay 7 days | PPHN | Major congenital anomaly |
|  |  | P-value | | | | | | | | | | | | | | | | |
| Gender | 0.004 | <.001 | <.001 | <.001 | <.001 | <.001 | <.001 | <.001 | 0.329 | <.001 | 0.808 | <.001 | <.001 | <.001 | <.001 | <.001 | <.001 | <.001 |
| Birthyear | <.001 | <.001 | 0.631 | <.001 | <.001 | 0.139 | <.001 | <.001 | 0.160 | 0.546 | <.001 | <.001 | <.001 | <.001 | <.001 | <.001 | <.001 | <.001 |
| Maternal age (cat) | <.001 | <.001 | <.001 | <.001 | <.001 | <.001 | <.001 | <.001 | <.001 | <.001 | <.001 | <.001 | <.001 | <.001 | <.001 | <.001 | <.001 | <.001 |
| Maternal age (cont) | <.001 | <.001 | <.001 | <.001 | <.001 | <.001 | <.001 | <.001 | <.001 | <.001 | <.001 | <.001 | 0.003 | <.001 | <.001 | <.001 | 0.001 | <.001 |
| Paternal age (cat) | <.001 | <.001 | <.001 | <.001 | <.001 | <.001 | <.001 | <.001 | <.001 | <.001 | <.001 | <.001 | <.001 | <.001 | <.001 | <.001 | 0.003 | <.001 |
| Paternal age (cont) | <.001 | <.001 | <.001 | <.001 | <.001 | <.001 | <.001 | <.001 | 0.009 | <.001 | <.001 | <.001 | 0.003 | <.001 | <.001 | <.001 | 0.022 | <.001 |
| Urbanity | <.001 | <.001 | <.001 | <.001 | <.001 | 0.882 | 0.047 | <.001 | <.001 | <.001 | 0.281 | <.001 | <.001 | <.001 | <.001 | <.001 | 0.044 | <.001 |
| Marital status | 0.002 | <.001 | <.001 | <.001 | <.001 | <.001 | <.001 | <.001 | <.001 | <.001 | <.001 | <.001 | <.001 | <.001 | <.001 | <.001 | 0.142 | <.001 |
| Maternal SES | <.001 | <.001 | <.001 | <.001 | <.001 | <.001 | <.001 | <.001 | <.001 | <.001 | <.001 | <.001 | <.001 | <.001 | <.001 | <.001 | <.001 | <.001 |
| Paternal SES | <.001 | <.001 | 0.061 | <.001 | <.001 | <.001 | <.001 | <.001 | <.001 | <.001 | <.001 | <.001 | <.001 | <.001 | <.001 | <.001 | <.001 | <.001 |
| Mother’s education | <.001 | <.001 | <.001 | <.001 | <.001 | <.001 | <.001 | <.001 | <.001 | <.001 | <.001 | <.001 | <.001 | <.001 | <.001 | <.001 | 0.012 | .11 |
| Father’s education | <.001 | <.001 | <.001 | <.001 | <.001 | <.001 | <.001 | <.001 | <.001 | <.001 | <.001 | <.001 | <.001 | 0.058 | <.001 | <.001 | 0.609 | .93 |
| Prev. births | <.001 | <.001 | <.001 | <.001 | <.001 | <.001 | <.001 | <.001 | <.001 | <.001 | <.001 | <.001 | <.001 | <.001 | <.001 | <.001 | 0.016 | <.001 |
| Smoking | <.001 | <.001 | <.001 | <.001 | <.001 | <.001 | <.001 | <.001 | <.001 | <.001 | <.001 | <.001 | <.001 | 0.015 | <.001 | <.001 | 0.346 | <.001 |
| Use of ART | <.001 | <.001 | <.001 | <.001 | <.001 | <.001 | <.001 | 0.185 | <.001 | <.001 | <.001 | <.001 | <.001 | <.001 | <.001 | <.001 | <.001 | <.001 |
| Maternal BMI | <.001 | <.001 | <.001 | <.001 | <.001 | <.001 | <.001 | <.001 | <.001 | <.001 | <.001 | <.001 | <.001 | <.001 | <.001 | <.001 | <.001 | <.001 |
| Pre-pregnancy diabetes | <.001 | <.001 | <.001 | <.001 | 0.008 | <.001 | <.001 | <.001 | <.001 | <.001 | <.001 | <.001 | <.001 | <.001 | <.001 | <.001 | <.001 | <.001 |
| Chronic diseases | <.001 | <.001 | <.001 | <.001 | <.001 | <.001 | <.001 | <.001 | <.001 | <.001 | <.001 | <.001 | <.001 | <.001 | <.001 | <.001 | <.001 | <.001 |
| Hospitalizations | <.001 | <.001 | <.001 | <.001 | <.001 | <.001 | <.001 | <.001 | <.001 | <.001 | <.001 | <.001 | <.001 | <.001 | <.001 | <.001 | <.001 | <.001 |
| Teratogens | <.001 | <.001 | <.001 | <.001 | 0.041 | <.001 | <.001 | <.001 | <.001 | <.001 | <.001 | <.001 | <.001 | <.001 | <.001 | <.001 | <.001 | <.001 |
| Anxiolytics and sedatives | <.001 | <.001 | <.001 | <.001 | <.001 | <.001 | 0.004 | 0.167 | <.001 | <.001 | 0.002 | <.001 | <.001 | <.001 | <.001 | <.001 | <.001 | <.001 |
| Antiepileptics | <.001 | <.001 | <.001 | <.001 | <.001 | <.001 | 0.171 | 0.837 | <.001 | <.001 | 0.013 | <.001 | <.001 | <.001 | <.001 | <.001 | <.001 | <.001 |
| Previous preterm births | <.001 | <.001 | <.001 | <.001 | 0.056 | <.001 | <.001 | <.001 | <.001 | <.001 | <.001 | <.001 | <.001 | <.001 | <.001 | <.001 | <.001 | <.001 |
| Previous hypertension | <.001 | <.001 | <.001 | 0.430 | 0.601 | <.001 | <.001 | <.001 | 0.231 | <.001 | <.001 | 0.001 | 0.309 | <.001 | <.001 | <.001 | 0.021 | <.001 |
| Previous gest. diabetes | <.001 | <.001 | <.001 | <.001 | <.001 | <.001 | 0.626 | <.001 | <.001 | <.001 | 0.136 | 0.056 | 0.288 | <.001 | <.001 | <.001 | <.001 | <.001 |

**Supplemental Table 2. Associations between individual SSRIs and pregnancy and neonatal complications.**

|  | | SSRI exposed | | SSRI exposed vs.  Women with depression but no antidepressants | |
| --- | --- | --- | --- | --- | --- |
|  | | N | % | OR (95% CI) | p |
| Pregnancy complications | | | | | |
| Hypertension | |  | | | |
|  | Fluoxetine | 271 | 11.1 | 1.09 (0.93, 1.27) | 0.29 |
|  | Citalopram | 723 | 10.1 | 1.003 (0.90, 1.12) | 0.95 |
|  | Paroxetine | 145 | 10.8 | 1.12 (0.91, 1.37) | 0.28 |
|  | Sertraline | 325 | 10.0 | 0.93 (0.81, 1.08) | 0.34 |
|  | Fluvoxamine | 15 | 7.1 | 0.57 (0.32, 0.997) | 0.05 |
|  | Escitalopram | 453 | 9.7 | 0.98 (0.87, 1.12) | 0.81 |
| Gestational diabetes | |  | | | |
|  | Fluoxetine | 415 | 17.1 | 1.18 (1.03, 1.35) | 0.02 |
|  | Citalopram | 1327 | 18.5 | 1.10 (1.007, 1.21) | 0.04 |
|  | Paroxetine | 218 | 16.3 | 1.15 (0.96, 1.39) | 0.12 |
|  | Sertraline | 589 | 18.2 | 1.02 (0.90, 1.15) | 0.77 |
|  | Fluvoxamine | 35 | 16.6 | 1.27 (0.82, 1.97) | 0.28 |
|  | Escitalopram | 948 | 20.4 | 1.15 (1.04, 1.28) | 0.007 |
| CS | |  | | | |
|  | Fluoxetine | 515 | 21.2 | 0.72 (0.64, 0.81) | <.001 |
|  | Citalopram | 1411 | 19.7 | 0.76 (0.70, 0.82) | <.001 |
|  | Paroxetine | 248 | 18.5 | 0.65 (0.55, 0.76) | <.001 |
|  | Sertraline | 655 | 20.2 | 0.81 (0.73, 0.90) | <.001 |
|  | Fluvoxamine | 45 | 21.3 | 0.59 (0.41, 0.85) | 0.005 |
|  | Escitalopram | 912 | 19.6 | 0.81 (0.74, 0.89) | <.001 |
| CS emergency or urgent | |  | | | |
|  | Fluoxetine | 206 | 8.5 | 0.997 (0.84, 1.18) | 0.97 |
|  | Citalopram | 689 | 9.6 | 0.93 (0.83, 1.03) | 0.15 |
|  | Paroxetine | 108 | 8.1 | 0.83 (0.66, 1.04) | 0.10 |
|  | Sertraline | 354 | 10.9 | 1.01 (0.89, 1.16) | 0.85 |
|  | Fluvoxamine | 22 | 10.4 | 1.42 (0.87, 2.32) | 0.16 |
|  | Escitalopram | 533 | 11.5 | 0.91 (0.81, 1.02) | 0.10 |
| Bleeding | |  | | | |
|  | Fluoxetine | 62 | 2.5 | 0.89 (0.67, 1.18) | 0.41 |
|  | Citalopram | 266 | 3.7 | 0.998 (0.85, 1.17) | 0.98 |
|  | Paroxetine | 35 | 2.6 | 0.80 (0.55, 1.15) | 0.23 |
|  | Sertraline | 132 | 4.1 | 0.96 (0.78, 1.18) | 0.68 |
|  | Fluvoxamine | 3 | 1.4 | 0.60 (0.19, 1.94) | 0.39 |
|  | Escitalopram | 263 | 5.7 | 1.21 (1.03, 1.41) | 0.02 |
| Late preterm | |  | | | |
|  | Fluoxetine | 119 | 4.9 | 0.91 (0.74, 1.13) | 0.40 |
|  | Citalopram | 331 | 4.6 | 0.82 (0.72, 0.95) | 0.007 |
|  | Paroxetine | 61 | 4.6 | 0.81 (0.60, 1.09) | 0.17 |
|  | Sertraline | 191 | 5.9 | 1.07 (0.90, 1.28) | 0.43 |
|  | Fluvoxamine | 18 | 8.5 | 1.58 (0.93, 2.68) | 0.09 |
|  | Escitalopram | 213 | 4.6 | 0.89 (0.75, 1.05) | 0.16 |
| Very preterm | |  | | | |
|  | Fluoxetine | 13 | 0.5 | 0.41 (0.23, 0.73) | 0.002 |
|  | Citalopram | 36 | 0.5 | 0.37 (0.25, 0.54) | <.001 |
|  | Paroxetine | 8 | 0.6 | 0.52 (0.26, 1.04) | 0.06 |
|  | Sertraline | 13 | 0.4 | 0.24 (0.12, 0.49) | <.001 |
|  | Escitalopram | 26 | 0.6 | 0.39 (0.25, 0.62) | <.001 |
| LGA | |  | | | |
|  | Fluoxetine | 75 | 3.1 | 0.91 (0.69, 1.18) | 0.46 |
|  | Citalopram | 236 | 3.3 | 0.93 (0.79, 1.11) | 0.44 |
|  | Paroxetine | 53 | 4.0 | 1.25 (0.91, 1.71) | 0.17 |
|  | Sertraline | 139 | 4.3 | 1.24 (1.001, 1.54) | 0.05 |
|  | Fluvoxamine | 5 | 2.4 | 0.41 (0.13, 1.33) | 0.14 |
|  | Escitalopram | 150 | 3.2 | 1.12 (0.91, 1.38) | 0.28 |
| SGA | |  | | | |
|  | Fluoxetine | 93 | 3.8 | 0.97 (0.77, 1.23) | 0.80 |
|  | Citalopram | 258 | 3.6 | 0.94 (0.80, 1.10) | 0.45 |
|  | Paroxetine | 40 | 3.0 | 0.81 (0.58, 1.13) | 0.21 |
|  | Sertraline | 115 | 3.5 | 0.95 (0.77, 1.18) | 0.66 |
|  | Fluvoxamine | 4 | 1.9 | 0.54 (0.20, 1.46) | 0.23 |
|  | Escitalopram | 149 | 3.2 | 0.83 (0.69, 1.01) | 0.06 |
| Low birth weight | |  | | | |
|  | Fluoxetine | 86 | 3.5 | 0.71 (0.56, 0.89) | 0.004 |
|  | Citalopram | 263 | 3.7 | 0.73 (0.62, 0.86) | <.001 |
|  | Paroxetine | 49 | 3.7 | 0.75 (0.54, 1.03) | 0.07 |
|  | Sertraline | 122 | 3.8 | 0.74 (0.60, 0.92) | 0.006 |
|  | Fluvoxamine | 6 | 2.8 | 0.58 (0.27, 1.25) | 0.16 |
|  | Escitalopram | 154 | 3.3 | 0.68 (0.56, 0.82) | <.001 |
| Very low birth weight | |  | | | |
|  | Fluoxetine | 17 | 0.7 | 0.61 (0.36, 1.02) | 0.06 |
|  | Citalopram | 29 | 0.4 | 0.30 (0.20, 0.47) | <.001 |
|  | Paroxetine | 8 | 0.6 | 0.56 (0.28, 1.13) | 0.11 |
|  | Sertraline | 10 | 0.3 | 0.20 (0.10, 0.43) | <.001 |
|  | Escitalopram | 20 | 0.4 | 0.30 (0.18, 0.51) | <.001 |
| *Neonatal complications* | | | | | |
| Apgar 1 min <7 | |  | | | |
|  | Fluoxetine | 253 | 10.4 | 1.71 (1.46, 2.00) | <.001 |
|  | Citalopram | 732 | 10.2 | 1.64 (1.47, 1.82) | <.001 |
|  | Paroxetine | 113 | 8.4 | 1.35 (1.09, 1.67) | 0.005 |
|  | Sertraline | 309 | 9.5 | 1.46 (1.27, 1.68) | <.001 |
|  | Fluvoxamine | 17 | 8.1 | 1.21 (0.68, 2.15) | 0.51 |
|  | Escitalopram | 431 | 9.3 | 1.42 (1.25, 1.61) | <.001 |
| Apgar 5 min <7 | |  | | | |
|  | Fluoxetine | 83 | 5.9 | 1.98 (1.53, 2.56) | <.001 |
|  | Citalopram | 362 | 6.5 | 2.11 (1.80, 2.48) | <.001 |
|  | Paroxetine | 54 | 5.8 | 1.90 (1.38, 2.60) | <.001 |
|  | Sertraline | 150 | 5.6 | 1.70 (1.38, 2.08) | <.001 |
|  | Fluvoxamine | 7 | 7.3 | 2.09 (0.94, 4.66) | 0.07 |
|  | Escitalopram | 222 | 5.4 | 1.74 (1.45, 2.08) | <.001 |
| Breathing problems | |  | | | |
|  | Fluoxetine | 205 | 8.4 | 1.70 (1.43, 2.01) | <.001 |
|  | Citalopram | 646 | 9.0 | 1.63 (1.45, 1.82) | <.001 |
|  | Paroxetine | 103 | 7.7 | 1.55 (1.23, 1.95) | <.001 |
|  | Sertraline | 296 | 9.1 | 1.53 (1.32, 1.77) | <.001 |
|  | Fluvoxamine | 18 | 8.5 | 2.01 (1.22, 3.32) | 0.006 |
|  | Escitalopram | 474 | 10.2 | 1.64 (1.44, 1.86) | <.001 |
| Monitoring (NCU) | |  | | | |
|  | Fluoxetine | 433 | 17.8 | 1.26 (1.11, 1.42) | <.001 |
|  | Citalopram | 1237 | 17.3 | 1.23 (1.14, 1.34) | <.001 |
|  | Paroxetine | 251 | 18.7 | 1.42 (1.21, 1.67) | <.001 |
|  | Sertraline | 612 | 18.9 | 1.38 (1.24, 1.53) | <.001 |
|  | Fluvoxamine | 44 | 20.9 | 1.48 (1.04, 2.12) | 0.03 |
|  | Escitalopram | 781 | 16.8 | 1.18 (1.07, 1.30) | 0.001 |
| Hospital stay ≥ 7 days | |  | | | |
|  | Fluoxetine | 205 | 8.5 | 0.81 (0.69, 0.96) | 0.02 |
|  | Citalopram | 560 | 7.9 | 0.84 (0.75, 0.94) | 0.002 |
|  | Paroxetine | 113 | 8.5 | 0.88 (0.71, 1.10) | 0.27 |
|  | Sertraline | 284 | 8.8 | 1.001 (0.87, 1.16) | 0.99 |
|  | Fluvoxamine | 28 | 13.4 | 1.42 (0.91, 2.21) | 0.12 |
|  | Escitalopram | 380 | 8.2 | 0.95 (0.83, 1.09) | 0.49 |
| Major congenital anomaly | |  | | | |
|  | Fluoxetine | 121 | 5.0 | 0.82 (0.66, 1.001) | 0.05 |
|  | Citalopram | 381 | 5.3 | 0.84 (0.73, 0.95) | 0.006 |
|  | Paroxetine | 80 | 6.0 | 0.93 (0.72, 1.21) | 0.59 |
|  | Sertraline | 170 | 5.2 | 0.83 (0.70, 0.99) | 0.04 |
|  | Fluvoxamine | 10 | 4.7 | 0.69 (0.31, 1.52) | 0.36 |
|  | Escitalopram | 241 | 5.2 | 0.75 (0.64, 0.88) | <.001 |

SSRI exposed: Two or more purchases of the specific SSRI anytime during pregnancy.

Adjusted for variables associated with exposure and outcome at p<0.1 (Supplemental Table 1).

Total N of exposed: Fluoxetine, n=2,433; Citalopram, n=7,159; Paroxetine, n=1,340; Sertraline, n=3,244; Fluvoxamine, n=211; Escitalopram, n= 4,651

**Supplemental Table 3.** Pregnancy and neonatal complications associated with first and second trimester SSRI use.

| **Outcome** | **SSRI use in first and second trimester**^a^  N=10,563 | | **Women with depression but no antidepressants**  N=19,625 | | **Women without depression or antidepressants**  **N=1,053,280** | | **SSRI use in first and second trimester**  ***vs.***  **Women with depression but no antidepressants** | | **SSRI use in first and second trimester *vs.***  **Women without depression or antidepressants** | |
| --- | --- | --- | --- | --- | --- | --- | --- | --- | --- | --- |
|  | **n** | **%** | **n** | **%** | **n** | **%** | **OR^b^ (95% CI**) | ***p*** | **OR^b^ (95% CI)** | ***p*** |
| *Pregnancy complications* | | | | | | |  |  |  |  |
| Hypertension | 1105 | 10.5 | 1790 | 9.1 | 81,630 | 7.8 | 0.996 (0.91, 1.09) | 0.925 | 1.06 (0.98, 1.14) | .14 |
| Gestational diabetes | 2091 | 19.8 | 2613 | 13.3 | 106,554 | 10.1 | 1.14 (1.05, 1.22) | <.001 | 1.18 (1.11, 1.25) | <.001 |
| Delivery mode (CS or vaginal) | 2139 | 20.3 | 4625 | 23.6 | 160,123 | 15.2 | 0.74 (0.70, 0.79) | <.001 | 1.15 (1.09, 1.21) | <.001 |
| CS emergency or urgent | 1154 | 10.9 | 1847 | 9.4 | 59,189 | 5.6 | 0.94 (0.86, 1.03) | 0.176 | 1.12 (1.05, 1.21) | 0.001 |
| Bleeding | 486 | 4.6 | 665 | 3.4 | 25,056 | 2.4 | 1.11 (0.98, 1.26) | 0.114 | 1.22 (1.10, 1.34) | <.001 |
| Late preterm | 556 | 5.3 | 1088 | 5.5 | 38,771 | 3.7 | 0.96 (0.85, 1.07) | 0.448 | 1.20 (1.09, 1.32) | <.001 |
| Very preterm | 64 | 0.6 | 284 | 1.4 | 5636 | 0.5 | 0.42 (0.30, 0.57) | <.001 | 0.90 (0.67, 1.20) | .46 |
| Large for gestational age, LGA | 358 | 3.4 | 597 | 3.0 | 30,340 | 2.9 | 0.99 (0.85, 1.14) | 0.863 | 1.07 (0.95, 1.20) | .29 |
| Small for gestational age, SGA | 353 | 3.3 | 818 | 4.2 | 32,090 | 3.1 | 0.87 (0.76, 1.0002) | 0.050 | 0.91 (0.81, 1.02) | .09 |
| Low birth weight | 405 | 3.8 | 1015 | 5.2 | 30,396 | 2.9 | 0.77 (0.68, 0.88) | <.001 | 1.07 (0.95, 1.20) | .25 |
| Very low birth weight | 52 | 0.5 | 252 | 1.3 | 4928 | 0.5 | 0.36 (0.25, 0.51) | <.001 | 0.80 (0.58, 1.10) | .16 |
| *Neonatal complications* | | | | | | |  |  |  |  |
| Apgar <7 1 min | 1154 | 11.0 | 1254 | 6.4 | 51,338 | 4.9 | 1.78 (1.63, 1.95) | <.001 | 1.96 (1.83, 2.10) | <.001 |
| Apgar <7 5 min | 575 | 6.6 | 455 | 3.3 | 11,509 | 2.0 | 2.26 (1.97, 2.60) | <.001 | 2.86 (2.59, 3.15) | <.001 |
| Breathing problems | 1113 | 10.5 | 1093 | 5.6 | 35,754 | 3.4 | 1.82 (1.66, 2.00) | <.001 | 2.23 (2.08, 2.40) | <.001 |
| Monitoring (NCU) | 2006 | 19.0 | 2809 | 14.3 | 97,343 | 9.2 | 1.33 (1.24, 1.43) | <.001 | 1.71 (1.62, 1.81) | <.001 |
| Hospital stay ≥7 days | 935 | 8.9 | 1778 | 9.2 | 53,842 | 5.1 | 0.95 (0.87, 1.04) | 0.291 | 1.47 (1.36, 1.59) | <.001 |
| Major congenital anomaly | 557 | 5.3 | 1194 | 6.1 | 41,123 | 3.9 | 0.81 (0.73, 0.90) | <.001 | 1.10 (1.01, 1.21) | .04 |

^a^At least one SSRI purchase during the first and second trimester.

^b^ Adjusted for variables associated with exposure and outcome at p<0.1 (Supplemental Table 1)

**Supplemental Table 4 .** Paternal use of SSRIs and pregnancy and neonatal complications.

| **Outcome** | **Paternal**  **SSRI use** ^a,b^   N=12,164 | | **No paternal SSRI use** ^a^  N=1,036436 | | **Paternal SSRI use**  ***vs.***  **No paternal SSRI use** | | |
| --- | --- | --- | --- | --- | --- | --- | --- |
|  | n | % | n | % | OR^c^ | 95% CI | *p* |
| *Pregnancy complications* | | | | | | | |
| Hypertension | 994 | 8.2 | 80283 | 7.7 | 1.05 | 0.98-1.13 | .14 |
| Gestational diabetes | 1409 | 11.6 | 104625 | 10.1 | 0.94 | 0.88 -1.004 | .07 |
| Delivery mode (CS or vaginal) | 1860 | 15.3 | 157559 | 15.2 | 0.995 | 0.95-1.04 | .85 |
| CS emergency or urgent | 805 | 6.6 | 58077 | 5.6 | 0.97 | 0.90-1.05 | .497 |
| Bleeding | 365 | 3.0 | 24553 | 2.4 | 1.03 | 0.92-1.15 | .57 |
| Late preterm | 442 | 3.6 | 38129 | 3.7 | 0.96 | 0.86-1.06 | .39 |
| Very preterm | 47 | 0.4 | 5570 | 0.5 | 0.69 | 0.51-0.94 | .02 |
| Large for gestational age, LGA | 334 | 2.8 | 29876 | 2.9 | 0.91 | 0.80-1.03 | .12 |
| Small for gestational age, SGA | 385 | 3.2 | 31536 | 3.0 | 1.03 | 0.93-1.15 | .53 |
| Low birth weight | 339 | 2.8 | 29897 | 2.9 | 0.96 | 0.85-1.07 | .44 |
| Very low birth weight | 36 | 0.3 | 4873 | 0.5 | 0.65 | 0.46-0.92 | .01 |
| *Neonatal complications* | | | | | | | |
| Apgar <7 1 min | 573 | 4.7 | 50531 | 4.9 | 0.94 | 0.86-1.02 | .14 |
| Apgar <7 5 min | 144 | 1.8 | 11307 | 2.0 | 0.86 | 0.73-1.03 | .095 |
| Breathing problems | 388 | 3.2 | 35182 | 3.4 | 0.84 | 0.75-0.93 | <.001 |
| Monitoring (NCU) | 1128 | 9.3 | 95746 | 9.2 | 0.95 | 0.89-1.02 | .13 |
| Hospital stay ≥7 days | 599 | 5.0 | 52981 | 5.1 | 0.96 | 0.88-1.04 | .32 |
| PPHN | 18 | 0.1 | 1226 | 0.1 | 1.19 | 0.75-1.89 | .46 |
| Major congenital anomaly | 489 | 4.0 | 40438 | 3.9 | 0.98 | 0.90-1.08 | .73 |

^a^ Including only pregnancies not exposed to maternal antidepressants or depression

^b^ Two or more SSRI purchases by the father during mother’s gestation

^c^ Adjusted for variables associated with exposure and outcome at p<0.1 (Supplemental Table 1).

**Supplemental Table 5.** Pregnancy and neonatal complications, comparisons between women with a diagnosis of depression but no antidepressant use during pregnancy and women unexposed to depression and antidepressants.

| **Outcome** | **Women with**  **depression**  **but no**  **antidepressants** | | **Unexposed** | | **Women with**  **depression**  **but no**  **antidepressants**  **vs.**  **Unexposed** | | | | | |
| --- | --- | --- | --- | --- | --- | --- | --- | --- | --- | --- |
|  | N=19,625 | | N=1,053,280 | | crude | | | adjusted^a^ | | |
|  | **n** | **%** | **n** | **%** | **OR** | **95% CI** | ***p*** | **OR** | **95% CI** | ***p*** |
| *Pregnancy complications* | | | | | | | | | | |
| Hypertension^b^ | 1790 | 9.12 | 81630 | 7.8 | 1.17 | 1.12-1.23 | <.001 | 1.06 | 1.01-1.12 | .03 |
| Gestational diabetes^c^ | 2613 | 13.31 | 106554 | 10.1 | 1.13 | 1.08-1.17 | <.001 | 1.04 | 0.99-1.09 | .13 |
| CS | 4625 | 23.58 | 160123 | 15.2 | 1.58 | 1.53-1.63 | <.001 | 1.54 | 1.49-1.60 | <.001 |
| CS  emergency  or urgent | 1847 | 9.41 | 59189 | 5.6 | 1.29 | 1.22-1.35 | <.001 | 1.19 | 1.13-1.26 | <.001 |
| Bleeding^d^ | 665 | 3.39 | 25056 | 2.4 | 1.02 | 0.94-1.11 | .59 | 1.10 | 1.01-1.19 | .03 |
| Late preterm^e^ | 1088 | 5.54 | 38771 | 3.7 | 1.52 | 1.42-1.61 | <.001 | 1.25 | 1.17-1.34 | <.001 |
| Very preterm^f^ | 284 | 1.45 | 5636 | 0.5 | 2.76 | 2.44-3.12 | <.001 | 2.15 | 1.86-2.48 | <.001 |
| LGA^g^ | 597 | 3.1 | 30340 | 2.9 | 1.12 | 1.03-1.22 | .006 | 1.08 | 0.99-1.18 | .10 |
| SGA^h^ | 818 | 4.17 | 32090 | 3.1 | 1.35 | 1.26-1.45 | <.001 | 1.03 | 0.96-1.12 | .40 |
| Low birth weight^i^ | 1015 | 5.17 | 30396 | 2.9 | 1.79 | 1.68-1.91 | <.001 | 1.39 | 1.29-1.49 | <.001 |
| Very low birth weight^j^ | 252 | 1.28 | 4928 | 0.5 | 2.79 | 2.45-3.19 | <.001 | 2.23 | 1.92-2.60 | <.001 |
| *Neonatal complications* | | | | | | | | | | |
| Apgar <7 1 min | 1254 | 6.4 | 51338 | 4.9 | 1.25 | 1.18-1.32 | <.001 | 1.10 | 1.04-1.18 | .002 |
| Apgar <7 5 min | 455 | 3.3 | 11509 | 2.0 | 1.59 | 1.45-1.75 | <.001 | 1.26 | 1.13-1.41 | <.001 |
| Breathing problems^k^ | 1093 | 5.6 | 35754 | 3.4 | 1.41 | 1.33-1.51 | <.001 | 1.23 | 1.15-1.31 | <.001 |
| Monitoring (NCU) | 2809 | 14.3 | 97343 | 9.2 | 1.55 | 1.48-1.61 | <.001 | 1.28 | 1.22-1.34 | <.001 |
| Hospital stay ≥7 days^l^ | 1778 | 9.2 | 53842 | 5.1 | 1.92 | 1.83-2.02 | <.001 | 1.54 | 1.46-1.63 | <.001 |
| PPHN^m^ | 55 | 0.3 | 1250 | 0.1 | 2.04 | 1.56-2.67 | <.001 | 1.80 | 1.34-2.41 | <.001 |
| Major congenital anomaly | 1194 | 6.1 | 41123 | 3.9 | 1.46 | 1.38-1.55 | <.001 | 1.36 | 1.28-1.45 | <.001 |

Women with depression but no antidepressants: Women diagnosed with depression or another psychiatric disorder related to depression or SSRI use from 1 year before pregnancy until discharge (≤3 weeks) from hospital after delivery, and with no maternal purchases of antidepressants or antipsychotics from 3 months before until the end of pregnancy.

Unexposed: No maternal prescriptions purchased for antidepressants or antipsychotics at any time prior to or during pregnancy nor any diagnoses of depression or psychiatric disorders related to depression or antidepressant use at any time prior to or during pregnancy until discharge (≤3 weeks) from hospital after delivery.

^a^Adjusted for variables associated with exposure and outcome at p<0.1 (Supplemental Table 1).

^b^Hypertension of pregnancy/ preeclampsia: ICD-10 O10-O16

^c^Gestational diabetes: ICD-10 O24.4; ICD-10 O24.9

CS, Cesarean section

^d^Bleeding during or after delivery (during delivery ICD-10 O67 (ICD-10 O67.0; 67.8; 67.9; after delivery ICD-10 O72 (ICD-10 O72.0-72.3)

^e^Late preterm 32-36+6 weeks

^f^Very preterm <32 weeks

^g^Large for gestational age (birth weight more than two standard deviations above national standards for sex and length of gestation)

^h^Small for gestational age (birth weight less than two standard deviations below national standards for sex and length of gestation)

^i^Low birth weight, <2,500g

^j^Very low birth weight (<1500 g)

^k^Neonatal breathing problems; ICD-10 P22

NCU; Neonatal Care or Intensive Care Unit

^l^Infant at hospital at 7 days of age

^m^Persistent pulmonary hypertension of the newborn; ICD-10 P29.31
